# Beyond Water: Deep Eutectic Solvents Enable Dye‐Sensitised Photocatalytic Hydrogen Production

**DOI:** 10.1002/cssc.70697

**Published:** 2026-05-08

**Authors:** Chiara L. Boldrini, Giorgia Salerno, Filippo M. Perna, Elizaveta Kozyr, Ottavia Bettucci, Vito Capriati, Lorenzo Mino, Alessandro Abbotto, Norberto Manfredi

**Affiliations:** ^1^ Department of Materials Science Solar Energy Research Center MIB‐SOLAR Consorzio CINMPIS and Consorzio INSTM University of Milano‐Bicocca Milano Italy; ^2^ Dipartimento di Farmacia–Scienze del Farmaco and Consorzio CINMPIS Università degli Studi di Bari “Aldo Moro” Bari Italy; ^3^ Department of Chemistry and Interdepartmental NIS Centre University of Torino Torino Italy

**Keywords:** carbazole, deep eutectic solvents, dye‐sensitised photocatalysis, hydrogen production, hydrophobic

## Abstract

This study reports the first demonstration of dye‐sensitised photocatalytic hydrogen evolution performed in deep eutectic solvents (DESs) as sustainable alternatives to conventional aqueous media. Platinum‐decorated TiO_2_ nanoparticles were sensitised with two carbazole‐based organic dyes featuring hydrophobic or hydrophilic peripheral substituents, enabling a systematic investigation of solvent–catalyst interfacial effects. Photocatalytic experiments were conducted in both hydrophobic and hydrophilic DESs, while water was retained exclusively as a proton source, thereby drastically reducing its overall consumption. Comprehensive physicochemical characterisation, including UV–visible spectroscopy, cyclic voltammetry, transmission electron microscopy, infrared spectroscopy, and nitrogen physisorption, was employed to elucidate structure–property relationships. Under visible‐light irradiation, DES‐based systems exhibited significantly enhanced hydrogen production rates, turnover numbers, and light‐to‐fuel efficiencies compared to aqueous benchmarks using the same sacrificial electron donor. Remarkably, the highest photocatalytic activity was observed for heterogeneous dye–DES combinations, revealing a counterintuitive trend with respect to dye–solvent affinity. These findings demonstrate that photocatalytic performance is critically governed by the interplay between dye functionalisation, DES polarity, and interfacial solvation, which collectively modulate charge transfer processes, surface wettability, and hydrogen evolution kinetics. Overall, this study establishes DESs as low‐cost, versatile, reusable, and environmentally benign reaction media, opening new avenues for solvent engineering in next‐generation photocatalytic hydrogen production.

## Graphical abstract

Dye‐sensitised photocatalytic H_2_ evolution is demonstrated for the first time in deep eutectic solvents (DESs). Carbazole‐based dyes with different hydrophobicity sensitise Pt@TiO_2_, revealing that heterogeneous dye–DES combinations maximise activity. Interfacial solvation and charge transfer govern enhanced hydrogen production under visible light.

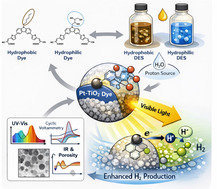

## 1 Introduction

In modern society, water is one of the most critical resources, fundamental both for the development of new technologies and for the survival of the human species. Its utilisation, particularly in the Global South, is often a source of social inequality, and, when scarce or absent, a concomitant driver of migration [1, 2, 3]. In an increasingly energy‐intensive world, water plays a central role not only in nutrition but also in energy sustainability [4, 5, 6, 7]. It can serve as an energy storage medium for renewable sources, enabling the production of zero‐CO_2_ solar fuels [8, 9] and hydrogen [10, 11, 12, 13]. However, the use of water as a hydrogen source inherently conflicts with the need to provide potable water to the population, thereby depriving a fundamental resource for survival precisely in those areas of the world where the deployment of new technologies should, in principle, be most accessible, owing to limited energy infrastructure and a rapidly growing demand. Furthermore, water employed in water‐splitting processes is most often highly purified, which increases process cost and further undermines overall sustainability. As a consequence, considerable research efforts have been devoted to identifying alternative media for hydrogen generation, ranging from seawater [14, 15, 16, 17, 18] to nonconventional reaction environments, with the dual aim of reducing freshwater consumption and maintaining reaction conditions more favourable to catalytic performance. Currently, the most mature technology for green hydrogen production is water electrolysis, which converts electrical energy, derived from renewable sources, into hydrogen and oxygen through electrolysers. This process is highly efficient and already viable on an industrial scale. Nonetheless, alternative systems must also be developed, as electrolysis requires large amounts of clean electricity, which at present accounts for only about one third of global electricity production. Moreover, the most efficient electrolysers—such as Proton Exchange Membrane devices—rely on precious and costly metal catalysts, including platinum, iridium or ruthenium. While these materials are compatible with acidic operating conditions, they significantly increase system costs, rendering the technology currently uncompetitive with hydrogen produced via methane steam reforming and fossil fuels (grey and blue hydrogen) [19]. An attractive alternative is offered by photocatalytic systems that mimic natural photosynthesis, directly converting water into hydrogen upon solar irradiation. These systems employ inorganic, organic, or hybrid semiconductors, in which distinct components fulfil complementary roles [20]. Among hybrid materials, titanium dioxide remains the most extensively investigated semiconductor. Its ability to drive water splitting under UV irradiation was first demonstrated in 1972 by Fujishima and Honda [21]. TiO_2_ is abundant and inexpensive; however, its wide bandgap severely limits its practical applicability, as UV radiation represents only ∼5% of the solar spectrum at the Earth's surface. To overcome this limitation, extensive efforts have focused on bandgap engineering through doping or sensitisation with organic dyes [22, 23, 24, 25]. As demonstrated over the years, the peripheral functionalization of these hybrid systems—and, crucially, the interactions between the functionalized surface and the surrounding medium—play a pivotal role in determining photocatalytic efficiency [23, 26, 27, 28]. In this context, the solvent should no longer be regarded as an inert and passive component, but rather as an active element capable of mediating reactivity, stabilising intermediates, and interacting dynamically with all components of the system. In this work, we propose replacing water as the bulk solvent for photocatalytic hydrogen generation, retaining it solely as a reagent and therefore in significantly reduced amounts, while substituting the reaction medium with an eco‐sustainable, reusable, and highly tuneable class of solvents: deep eutectic solvents (DESs). Much like water, DESs can be considered ‘living solvents’, as they are not merely passive media but actively participate in the reaction environment through extended hydrogen‐bond networks, strong solvation effects, and specific interactions with catalysts, electrodes, and photosensitisers [29]. These features enable DESs to modulate reaction pathways, stabilise charged or radical intermediates [30], and influence interfacial phenomena at semiconductor surfaces. DESs have already demonstrated remarkable versatility across multiple areas of chemistry, including synthesis, catalysis, and energy‐related applications [31, 32, 33]. To the best of our knowledge, this study reports the first example of a DES employed as the solvent medium for photocatalytic hydrogen generation.

**FIGURE 1:**
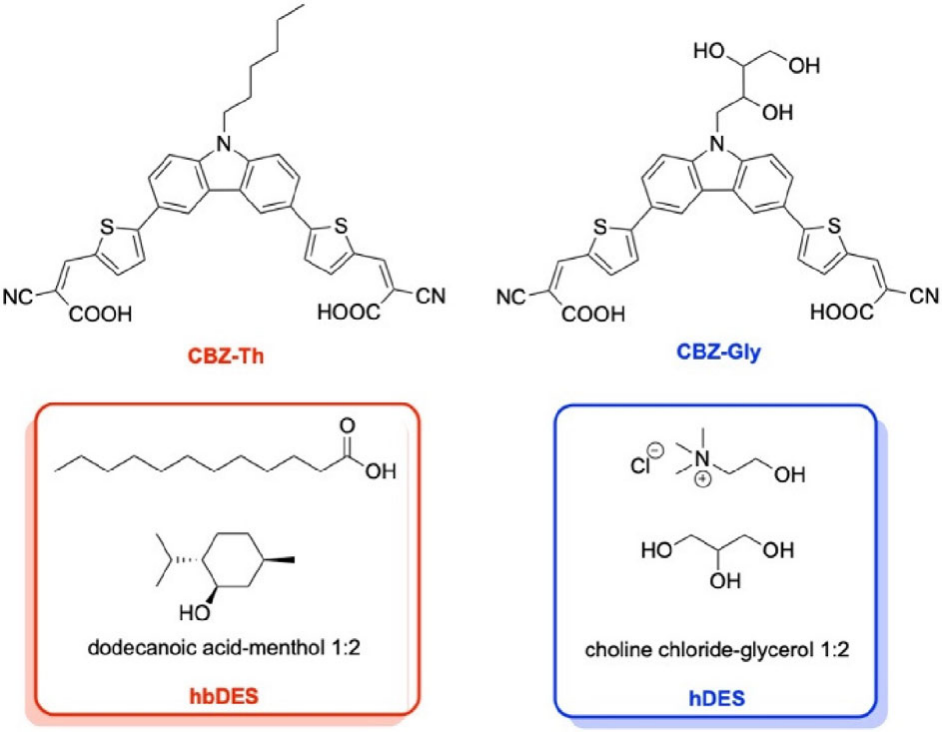
Structures of investigated sensitisers and selected DES mixtures exploited in this work.

To elucidate the role of the solvent at the interface with the hybrid dye‐sensitised nanoparticles, we investigated two sensitisers bearing different peripheral functionalization deliberately designed to promote distinct interactions with the surrounding medium. Specifically, two carbazole (CBZ)‐based dyes were selected, functionalized with either an alkyl chain or a glycolic pendant, acting as hydrophobic (CBZ‐Th) and hydrophilic (CBZ‐Gly) sensitisers, respectively [34, 35]. The carbazole core was chosen due to its outstanding performance in photocatalytic hydrogen generation, as previously demonstrated by our group in comparison with other heteroaromatic scaffolds. The sensitisers were evaluated in two distinct DES systems: dodecanoic acid:menthol 1:2, a hydrophobic DES (hbDES) [36], and choline chloride:glycerol 1:2 (hDES), a hydrophilic DES (Figure 1). Both DESs are composed of inexpensive, widely available, and nontoxic components, making them particularly attractive and sustainable alternatives to water as reaction media.

The deliberate comparison between hydrophilic and hydrophobic DESs was guided by our previous studies on sustainable dye‐sensitised solar cells (DSSCs) [37, 38, 39, 40], where DES polarity and hydrogen‐bonding capability were shown to exert a profound influence on interfacial organisation, dye regeneration efficiency, and charge–transfer processes. Building on this established framework, we extend the same conceptual approach to photocatalytic hydrogen generation, where analogous interfacial and solvation effects are expected to be equally critical. In this context, hDESs are anticipated to promote stronger interactions with polar functional groups of the dye, the TiO_2_ surface, and water molecules acting as reagents, potentially facilitating proton‐coupled electron‐transfer processes. Conversely, hbDESs may induce different solvent structuring, water distribution, and dye–surface interactions at the interface. A direct comparison between these two solvent environments, therefore, provides a rational strategy to probe how solvent polarity and microstructural organisation modulate photocatalytic performance, ultimately shedding light on the active role of DESs as tuneable, bio‐inspired reaction media in hybrid photocatalytic systems.

## 2 Experimental Section

### 2.1 Materials and Methods

The two dyes, CBZ‐Th and CBZ‐Gly, were synthesised according to the previously reported procedure [34, 35]. To perform photocatalytic experiments, TiO_2_ (CAS: 13 463‐67−7, cat. n.: 718 467), dodecanoic acid (CAS: 143‐07−7, cat. n.: L4250), menthol (CAS: 89‐78−1, cat. n.: 63 670), choline chloride (CAS: 67‐48−1, cat n.: C7017), glycerol (CAS: 56‐81−5, cat. n.: G7893), hexachloroplatinic(IV) acid (CAS: 26 023‐84−7, cat. n.: 254 029) ethanol (CAS: 64‐17−5, cat. n.: 1.00983), and water (CAS: 7732‐18−5, cat. n.: 00612) were purchased from Merck and used without any further purification unless otherwise noted.

#### 2.1.1 DESs Characterisation

The thermogravimetric analysis (TGA) of the DES mixtures was carried out using a TGA/DSC1 STARe system in the temperature range 30°C–500°C, with a heating rate of 10°C/min and a constant air flow of 50 mL/min. The viscosity was measured by calculating the mean value of three independent measurements using a Atago Visco‐895 digital viscometer at 18°C ± 1°C, with an accuracy of ± 12 mPa·s for hDES and of ± 3.6 for hbDES (the different viscosities required a different setup).

#### 2.1.2 Optical and Electrochemical Measurements

UV–visible (UV–vis) diffuse reflectance measurements of the dyes adsorbed on the Pt@TiO_2_ catalytic materials were acquired over 350–800 nm using a Jasco V‐770 double‐beam spectrophotometer, equipped with a Jasco ISN‐470 integrating sphere. The parameters used were UV–vis bandwidth: 1 nm, NIR bandwidth: 4 nm, UV–vis response: 0.96 s, NIR response: 3.84 s, scan speed: 100 nm/min.

Cyclic voltammetry (CV) was carried out using a Bio‐logic SP‐240 potentiostat in a three‐electrode electrochemical cell. The scan rate was set at 20 mV s^−1^, and the measurements were performed in a 0.1 M tetrabutylammonium tetrafluoroborate (TBABF_4_) solution in CH_3_CN as a supporting electrolyte under nitrogen. The working electrode was a glassy carbon pin with 1.6 mm diameter (surface area = 0.08 cm^2^) in the case of DESs and a dye‐sensitised TiO_2_/FTO substrate working electrode (surface area = 0.196 cm^2^) in the case of dyes. The counter and pseudo‐reference electrodes were a Pt wire and an Ag/Ag^+^ TBAP in CH_3_CN (0.1 M tetrabutylammonium perchlorate and 0.01 M AgNO_3_ in acetonitrile (ACN)). The Ag/Ag^+^ pseudo‐reference electrode was calibrated by adding ferrocene (10^−3^ M, Fc) to the test solution after each measurement. The Pt wire was sonicated for 15 min in deionised water, washed with 2‐propanol, and cycled for 50 times in 0.5 M H_2_SO_4_ before use.

#### 2.1.3 Preparation and Characterisation of TiO_2_/Pt Nanoparticles

Platinization of commercial TiO_2_ Degussa P25 was done using a modified photodeposition method previously reported in the literature [41]. TiO_2_ Degussa P25 (500 mg) was suspended in a solution of H_2_O (50 mL) and EtOH (50 mL) containing 10.5 mg of H_2_PtCl_6_ to reach a final metal loading of 1.0 wt%. After stirring for 1 h in the dark, the suspension was irradiated with a 250 W medium‐pressure lamp for 4 h. Decorated nanoparticles were collected by centrifugation (10 min, 11 000 rpm), washed with ethanol (3 × 10 min, 11 000 rpm), and dried under vacuum at room temperature overnight. The obtained nanoparticles were characterised combining the following techniques.

Fourier transform infrared spectroscopy (FTIR) spectra were recorded in transmission mode at a resolution of 2 cm^−1^ using a Bruker INVENIO FTIR spectrometer equipped with a DTGS detector. The samples were pressed into self‐supporting pellets and mounted in a custom‐designed quartz IR cell with KBr windows, allowing in situ measurements. Before CO adsorption the samples were outgassed for 1 h at 150°C and then reduced in H_2_ at the same temperature. Each spectrum represents the average of 64 scans, and it is normalized to the optical density (mg cm^−2^) of the pellet.

Transmission electron microscopy (TEM) measurements were carried out using a Tecnai BioTwin Spirit microscope. Samples were prepared by suspending the material in EtOH and dispersing it onto commercial TEM grids coated with a carbon film. The images were processed in ImageJ (v. 1.54g, National Institutes of Health, Bethesda, MD, USA) code [42].

BET surface area was obtained by N_2_ adsorption at 77 K using an ASAP2020 instrument (Micromeritics). All samples were outgassed at 120°C before the adsorption experiments.

#### 2.1.4 Dye Loading of CBZ‐Based Sensitisers on TiO_2_/Pt Nanoparticles

Dye staining (Dye@TiO_2_/Pt) was achieved as described in our previous paper where optimisation of the loading was done [26, 34]. 100 mg of TiO_2_/Pt nanoparticles were suspended in 10 mL of dye solution (0.1 mM in 9.5 mL of EtOH and 0.5 mL of DMSO) for 12 h in the dark. Then the Dye@TiO_2_/Pt nanocomposite was collected by centrifugation (10 min, 11 000 rpm), washed with EtOH (2 × 10 min, 11 000 rpm), and dried under vacuum at room temperature overnight in the dark to prevent photodegradation. The concentration of the dyes in the supernatant liquid was measured by UV–vis spectroscopy, confirming the quantitative loading of dyes on the TiO_2_/Pt material.

#### 2.1.5 Photocatalytic Hydrogen Generation Measurements

The Dye@TiO_2_/Pt nanomaterials have been tested for H_2_ production modifying a procedure described in the literature. Briefly, the reactor is a glass bottle with a PTFE septum on top (volume of ∼35 mL). 10 mg of the Dye@TiO_2_/Pt was suspended into 10 mL of 10% v/v aqueous solution of triethanolamine (TEOA) neutralised with HCl to reach a pH = 6.7 for the control experiments, or in 10 mL of 10% v/v solution of TEOA in the appropriate DES. After purging with Ar (15 mL min^−1^) for 30 min, the reactor containing the suspension is irradiated with a white source (Quantum Design) mounting a 300 W Xe lamp, a cutoff filter at λ = 400 nm and a heat adsorbing filter removing all photons with λ > 800 nm. Irradiance was measured by a calibrated photodiode as *P*_tot_ = 750 W m^−2^ in the Vis range (400–800 nm). The irradiated area (*A*_irr_) was of 19.2 cm^2^. The concentration of H_2_ in the gas phase of the reactor has been quantified using a Agilent 6850 gas‐chromatograph, with Ar as carrier (flow rate 25 mL min^−1^), equipped with a TCD detector, connected to a molecular sieve 5 Å column (2 m × 2 mmID, temperature 70°C). Argon mixtures with 100 and 10 000 ppm of H_2_ were used for calibration. H_2_ concentration was quantified by manual injection (100 µL volume with a Hamilton 1710RN gas‐tight syringe) every 20 min. The performances of the photocatalysts have been reported in terms of overall H_2_ productivity. Turnover numbers (TONs) were calculated as (2 × overall H_2_ amount)/(dye loading).

Light‐to‐fuel efficiency (LFE) was calculated as

$$\mathrm{LFE}=\frac{F_{H_{2}}\times {\Delta H}_{H_{2}}^{0}}{S\times A_{\mathrm{irr}}}$$

where *F*_H__2_ is the flow of H_2_ produced (expressed in mol s^−1^), Δ*H* ^0^_H__2_ is the enthalpy associated with H_2_ combustion (285.8 kJ mol^−1^), *S* is the total incident light irradiance, in 400–800 nm ranges (expressed in W cm^−2^), and *A*_irr_ is the irradiated area (expressed in cm^2^).

## 3 Results and Discussion

### 3.1 Materials Characterisation

**FIGURE 2:**
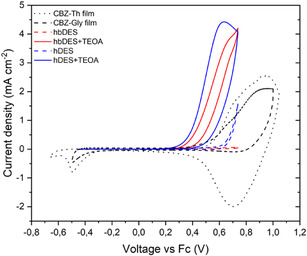
CV scans of the DES mixtures with (solid line) or without (dashed line) TEOA compared with sensitisers adsorbed onto a TiO_2_ film performed in an ACN solution with 0.1 M TBABF_4_ as a supporting electrolyte at a scan rate of 20 mV s^−1^.

Considering the potential role of the DES mixture as an active component in the photocatalytic process, we investigated the electrochemical behaviour of the DES systems by CV in solution to evaluate whether the oxidation potentials of the individual DES constituents are compatible with their possible function as sacrificial electron donors (SEDs). The results, shown in Figure 2, compare the oxidation profiles of the pristine DES mixtures (dashed coloured lines), the reference CBZ‐Th and CBZ‐Gly dyes absorbed on a TiO_2_ mesoporous film deposited on an FTO electrode (dotted and dashed black line, respectively), and the DES mixture containing 10% w/w TEOA as an external SED. All CV measurements were carried out in CH_3_CN solution with 0.1 M TBABF_4_ as the supporting electrolyte.

**FIGURE 3:**
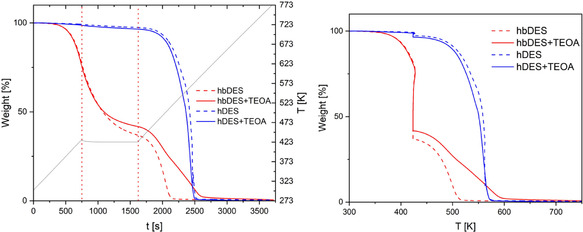
TGA in the temperature range 30°C–500°C, with a heating rate of 10°C/min and a constant air flow of 50 mL/min of DES mixtures with (solid line) or without (dashed line) TEOA. The grey line represents the heating ramp.

The CV scans clearly indicate that the investigated DESs are not able to reduce the radical cation of the sensitisers adsorbed onto the TiO_2_ surface of the photocatalyst. Indeed, the pristine DES mixtures remain electrochemically stable within the potential window in which oxidation of the sensitiser occurs. On this basis, the addition of an external SED is required to enable efficient dye generation. Upon incorporation of TEOA, an additional oxidation feature appears at a significantly lower anodic potential, which is compatible with the reduction of the dye radical cation. TEOA is a common component in DES formulations [43], where it typically acts as a hydrogen bond acceptor in combination with either choline chloride or long‐chain fatty acids. We therefore anticipate that the introduction of a limited amount of TEOA does not substantially perturb the intrinsic physicochemical properties of the DESs. This assumption is further supported by TGA, which reveals comparable thermal behaviour for both DES mixtures in the presence and absence of TEOA (Figure 3). In photocatalytic hydrogen generation, the availability of a proton source is a crucial parameter, as proton reduction is a prerequisite for molecular hydrogen evolution. The water content in the DES mixture was quantified by Karl Fisher titration and found to be ≈1% w/w, which is sufficient to provide an adequate concentration of protons in the reaction medium (Table S1 in the Supporting Information). In addition to water content, pH plays a key role in determining proton availability, and therefore directly influences the kinetics of photocatalytic hydrogen reduction. Accordingly, the pH of both pristine DESs was measured and found to be slightly acidic, with values in the range of 5–6, in good agreement with literature reports [44, 45]. Upon addition of TEOA, the pH of the DES mixture shifts to alkaline values, ranging ≈8 to nearly 10 (Table S1, the Supporting Information).

To further assess the DES nature of the modified mixtures, the viscosity was measured before and after the addition of the TEOA. The resulting values (Table S1, the Supporting Information) confirm that, upon addition of the TEOA, this parameter was not particularly affected, showing values between 500 ± 12 and 560 ± 12 mPa·s for hDES [46] and hDES + TEOA, respectively, and 37.5 ± 3.6 and 90.0 ± 3.6 mPa·s for hbDES [47] and hbDES + TEOA.

**FIGURE 4:**
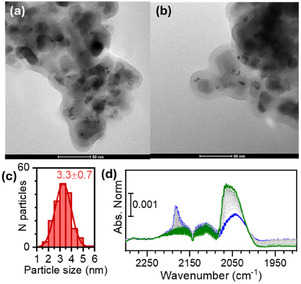
TEM images (a and b) and corresponding Pt particle size distribution (c) for the Pt@TiO_2_ sample. (d) IR spectra of CO adsorbed on Pt@TiO_2_, previously reduced at 150°C, at increasing contact times. The blue curve corresponds to the first measurement after the introduction of CO at 35 mbar, while the green spectrum was recorded after 1 h of exposure. The spectrum of the sample before dosing CO was subtracted from all the spectra.

The Pt‐decorated titania photocatalyst was prepared by photodeposition of metallic Pt nanoparticles onto Degussa P25 TiO_2_. Briefly, a suspension of TiO_2_ nanoparticles in a 1:1 v/v of water/EtOH mixture containing an appropriate amount of H_2_PtCl_6_ as the platinum precursor was irradiated with a 250 W Hg lamp, following a modified literature procedure [41]. Under UV irradiation, photogenerated electrons reduce Pt^4+^ ions, leading to the formation and deposition of Pt(0) nanoparticles on the TiO_2_ surface, thereby generating a catalytically active composite. As a first step in the characterisation of the Pt@TiO_2_ nanoparticles, N_2_ adsorption measurements at 77 K were performed (Figure S2). The analysis yielded a BET specific surface area of 55 m^2^ g^−1^ and revealed the absence of significant porosity, in good agreement with literature values reported for TiO_2_ P25 [48]. The morphology and dispersion of the Pt nanoparticles were subsequently investigated by TEM. Representative TEM images (Figure 4a,b) show TiO_2_ P25 particles with the characteristic irregular bipyramidal morphology, predominantly exposing {101} facets, which are known to possess the lowest surface energy [49]. Well‐dispersed Pt nanoparticles are clearly visible on TiO_2_ surface, displaying a narrow particle size distribution with an average diameter of 3.3 ± 0.7 nm (Figure 4c).

To gain deeper insight into the surface properties and coordination environment of the metallic phase, IR spectroscopy using CO as a probe molecule was employed. As shown in Figure 4d, a band at 2183 cm^−1^ is observed and attributed to CO interacting with the Lewis acidic sites of the {101} facets of anatase TiO_2_ [49]. In addition, a broad absorption feature at lower wavenumbers is detected, which can be deconvoluted into at least two components. The band centred at 2065 cm^−1^ is assigned to CO adsorbed on undercoordinated Pt^0^ atoms located at edge sites [50], while the component at 2040 cm^−1^ is associated with highly undercoordinated metallic Pt sites at more defective positions, such as corners and kinks [50]. This distribution of surface sites is fully consistent with the small particle size and high dispersion of Pt nanoparticles inferred by TEM analysis.

The resulting Pt@TiO_2_ material was subsequently sensitised with both dyes, affording a dye loading of 10 μmol g^−1^, in line with previous studies performed under optimised conditions [26]. Successful absorption of the sensitisers onto the photocatalyst surface was confirmed by UV–vis diffuse reflectance spectroscopy, with the corresponding spectra reported in Figure S1.

### 3.2 Photocatalytic H_2_ Generation

Photocatalytic H_2_ evolution was then investigated across four distinct dye–DES systems, obtained by systematically combining hydrophobic and hydrophilic sensitisers (CBZ‐Th@TiO_2_/Pt and CBZ‐Gly@TiO_2_/Pt) with hydrophobic and hydrophilic DESs. Specifically, the following four combinations were tested: (i) hydrophobic dye in hbDES, (ii) hydrophobic dye in hDES, (iii) hydrophilic dye in hbDES, and (iv) hydrophilic dye in hDES. This matrix‐like experimental design enabled a direct assessment of the interplay between dye functionalisation and solvent polarity.

**FIGURE 5:**
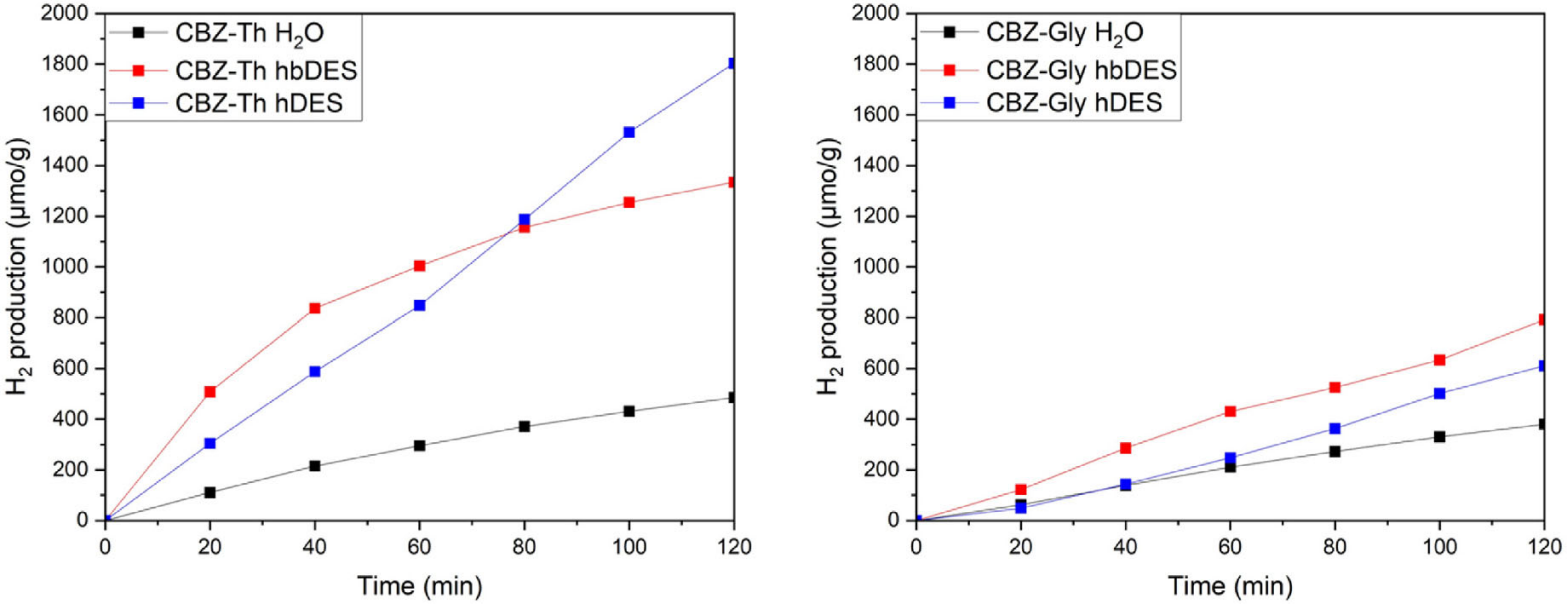
H_2_ production measured using the CBZ‐Th@TiO_2_/Pt photocatalysts (left) and CBZ‐Gly@TiO_2_/Pt (right) in hydrogen production experiments from TEOA 10 v/v% aqueous solution at pH = 6.7 (black line), in hbDES + TEOA (red line) and in hDES + TEOA (blue line) under irradiation with Vis light (400 nm < λ < 800 nm).

Photocatalytic H_2_ evolution in these four dye–DES systems was compared with benchmark experiments conducted in conventional aqueous media using TEOA as a SED. Visible‐light irradiation was granted by filtering a xenon lamp equipped with a band‐pass filter (400 nm < λ < 800 nm). Under these conditions, no H_2_ evolution was detected either for bare Pt/TiO_2_ or for CBZ‐sensitised photocatalysts in both hbDES and hDES in the absence of TEOA, confirming the necessity of dye sensitisation and SED. The time‐dependent H_2_ evolution profiles are shown in Figure 5 while the corresponding TONs and LFE calculated after 2 h of irradiation (LFE_2_) are summarised in Table 1 [51, 52].

**TABLE 1:** Photocatalytic performances in H_2_ production measured using the CBZ‐Th@TiO_2_/Pt photocatalysts and CBZ‐Gly@TiO_2_/Pt in hydrogen production experiments from TEOA 10 v/v% aqueous solution at pH = 6.7, in hbDES + TEOA and in hDES + TEOA under irradiation with Vis light (400 nm < λ < 800 nm).

**CBZ‐Th@TiO**_**2**_ **/Pt**			**CBZ‐Gly@TiO**_**2**_ **/Pt**		
**H**_**2**_ **production,** μmol/g	**TON**_**2**_	**LFE**_**2**_, %	**H**_**2**_ **production,** μmol/g	**TON**_**2**_	**LFE**_**2**_, %
**H**_**2**_ **O**	484.7	32.3	0.04	375.4	25.0	0.03
**hbDES**	1334.2	88.9	0.14	791.5	52.8	0.08
**hDES**	1803.5	120.2	0.16	610.7	40.7	0.06

The relatively short duration of the photocatalytic experiments is primarily attributable to the mildly alkaline nature of the DES mixtures in the presence of TEOA (see above), which promotes partial dye desorption from the catalytic composite [53]. Despite this limitation, the H_2_ evolution efficiencies achieved in both hbDES and hDES containing TEOA are remarkably high, substantially outperforming those obtained in water under identical conditions with the same SED.

Strikingly, for both sensitisers, the trend in photocatalytic H_2_ production is opposite to that expected on the basis of dye–DES affinity. Specifically, the hydrophobic dye exhibits higher H_2_ evolution in the hydrophilic DES, while the hydrophilic dye performs better in the hydrophobic DES. This counterintuitive behaviour indicates that heterogeneous dye–DES combinations give rise to the most efficient photocatalytic systems. When overall photocatalytic performance is considered, H_2_ production is consistently higher for CBZ‐Th than for CBZ‐Gly, with enhancement factors ranging from ≈1.6 in hbDES + TEOA to nearly 3 in hDES + TEOA. This behaviour contrasts sharply with our previous observations in dye‐sensitised solar cells, where optimal performance was achieved when the hydrophilicity or hydrophobicity of the sensitiser matched that of the DES, owing to favourable dye–solvent interactions [35]. In the present photocatalytic systems, this discrepancy can be rationalised by considering differences in dye organisation and orientation at the catalyst surface. As previously reported, peripheral functional groups capable of coordinating or interacting with the semiconductor surface strongly influence dye packing, surface arrangement, and interfacial charge–transfer pathways, all of which are critical for efficient hydrogen production [26, 54].

However, from the DES perspective, the best performance is observed in heterogeneous pairs. This behaviour can be ascribed to the combined effect related to the environment created by the DES at the interface with the functionalized catalyst. The affinity between the wettability induced on the surface of the catalytic system by dye absorption and the reaction environment tends to enhance the formation and detachment of hydrogen microbubbles from the surface of the material itself, thus making the catalytic sites more available [55, 56, 57]. However, these studies were performed on materials with a very different structure and always in an aqueous environment, while their consequences in DES‐based photocatalytic systems remain largely unexplored.

Similarly, from the catalytic materials perspective, the presence of long alkyl chains is well documented and known to reduce the aggregation of sensitisers on the surface, improving charge transfer towards the semiconductor [23, 58, 59]. The presence of a highly hydrophobic surface also reduces dye desorption from the surface of the catalytic material in the presence of water.

Another remarkable aspect that highlights the importance of the specific interactions between DES and the sensitised catalyst is the fact that hydrogen production is greater whenever a DES is used rather than water. This is even more relevant considering that the pH of the DESs explored in this work is alkaline due to the presence of TEOA, and therefore, the proton availability is significantly lower than that in water. This can only be explained by the specific properties of DESs. Interestingly, a similar behaviour has been observed in other transformations carried out in DESs. For instance, sulfonamide synthesis proceeds with enhanced efficiency when reactants are poorly soluble in the DES, shifting the reaction site from the bulk phase to the DES‐substrate interface [60]. Under these conditions, the reaction predominantly occurs at the DES‐substrate interface rather than in the bulk phase. Such interfacial microenvironments promote higher reaction rates due to unique solvent structuring and localised reactant concentration. Likewise, the photocatalytic system studied here likely benefits from similar interfacial effects, where the heterogeneous sensitised catalyst‐DES combination facilitates more efficient charge–transfer and turnover. Notably, DES have demonstrated to be a suitable platform in many different reactions, acting not only as a solvent but also as an active component in catalytic processes [33]. The capability of DESs to modulate catalytic behaviour arises from their highly customizable structure. By adjusting their components, it is possible to control many properties such as polarity, acidity, viscosity, interfacial and solvation properties, facilitating the engineering of task‐specific solvents that could optimise both catalytic activity and selectivity. This allows to express very peculiar properties at the interface, enhancing reactivity in many different cases.

## 4 Conclusion

In this work, we report for the first time the use of DESs as supporting media for dye‐sensitised photocatalytic hydrogen production. Platinum‐decorated TiO_2_ nanoparticles were sensitised with two carbazole‐based organic dyes bearing either hydrophobic (CBZ‐Th) or hydrophilic (CBZ‐Gly) peripheral functionalities and evaluated in both hydrophobic and hydrophilic DES formulations in the presence of TEOA as a SED. Comprehensive physicochemical characterisation of both the catalytic materials and the DESs was performed, followed by photocatalytic testing of hydrogen generation under visible‐light irradiation. The DES‐based systems consistently and markedly outperformed conventional aqueous media, achieving up to a threefold enhancement in hydrogen evolution rates, TONs, and light‐to‐fuel efficiencies. These results establish DESs as a viable and sustainable alternative to water for dye‐sensitised photocatalytic hydrogen production and represent a conceptual advance in the design of solar‐driven fuel‐generating systems. Notably, by replacing bulk water with DESs and confining its role to that of a proton source, the overall water demand is significantly reduced, addressing a critical sustainability challenge associated with both conventional photocatalytic and electrolytic hydrogen production technologies. The intrinsic properties of DESs—including low cost, wide availability, negligible volatility, facile preparation from benign components, chemical stability, and reusability—further enhance the environmental and technological appeal of this approach.

Beyond performance metrics, this study demonstrates that photocatalytic activity is not governed solely by the intrinsic properties of the sensitiser or the semiconductor, but rather by the complex interplay at the solvent–catalyst interface. The polarity, hydrogen‐bonding network, and wettability imparted by the DES strongly influence dye organisation, interfacial charge separation, and the kinetics of proton reduction as well as hydrogen bubble nucleation and detachment from the surface of the catalyst. The superior performance observed for heterogeneous dye–DES combinations highlights the crucial role of interfacial solvation and microenvironmental structuring in facilitating efficient charge transfer while maintaining a high density of accessible catalytic sites.

Importantly, all DES‐based systems outperform their aqueous counterparts, underscoring solvent engineering as a powerful and largely underexplored strategy to boost photocatalytic efficiency without relying on critical raw materials or complex and costly material modifications. Overall, this work positions DESs as tuneable, low‐impact, and potentially scalable media for sustainable photocatalysis, providing foundational insights to guide the rational development of next‐generation systems for solar hydrogen production and broader energy conversion applications.

## Supporting Information

Additional supporting information can be found online in the Supporting Information section.

## Funding

This study was supported by Ministero dell’Ambiente e della Sicurezza Energetica (RSH2A_000004, F57G25000080006), Ministero dell’Università e della Ricerca (2022KMS84P), NextGenerationEU (CN00000023).

## Conflicts of Interest

The authors declare no conflicts of interest.

## Supporting information

Supplementary Material

## Data Availability

The data that supports the findings of this study are available in the supplementary material of this article.
