# Supplementary material for: Beyond Water: Deep Eutectic Solvents Enable Dye‐Sensitised Photocatalytic Hydrogen Production
Source: ChemSusChem. 2026 May 8;19(9):e70697. doi: 10.1002/cssc.70697 (PMC13154723; doi:10.1002/cssc.70697)
Supplement: Supplementary file 1 — Supplementary Material [file CSSC-19-e70697-s001.pdf]

Beyond Water: Deep Eutectic Solvents Enable Dye-Sensitised Photocatalytic Hydrogen Production

Supporting Information

Chiara L. Boldrini, Giorgia Salerno, Filippo M. Perna, Elizaveta Kozyr, Ottavia Bettucci, Vito Capriati, Lorenzo Mino, Alessandro Abbotto,* and Norberto Manfredi*

Table of content

Table S1. Water content measured using the Karl-Fisher technique, viscosity and pH of pure DESs and DESs containing TEOA. 3

Figure S1. UV-Vis diffuse reflectance measurements of the dyes adsorbed on Pt@TiO_2. 3

Figure S2. Adsorption–desorption N_2 isotherms measured at 77 K for Pt@TiO_2 sample. 4

Table S1. Water content measured using the Karl-Fisher technique, viscosity and pH of pure DESs and DESs containing TEOA.

	pH	H_2O content	Viscosity at 18 °C ± 1 °C
		%wt	mPa·s
hbDES	5.5	0.71	37.5 ± 3.6
hDES	6.6	1.06	494 ± 12
hbDES + TEOA	8.4	0.61	90 ± 3.6
hDES + TEOA	9.7	1.09	561 ± 12

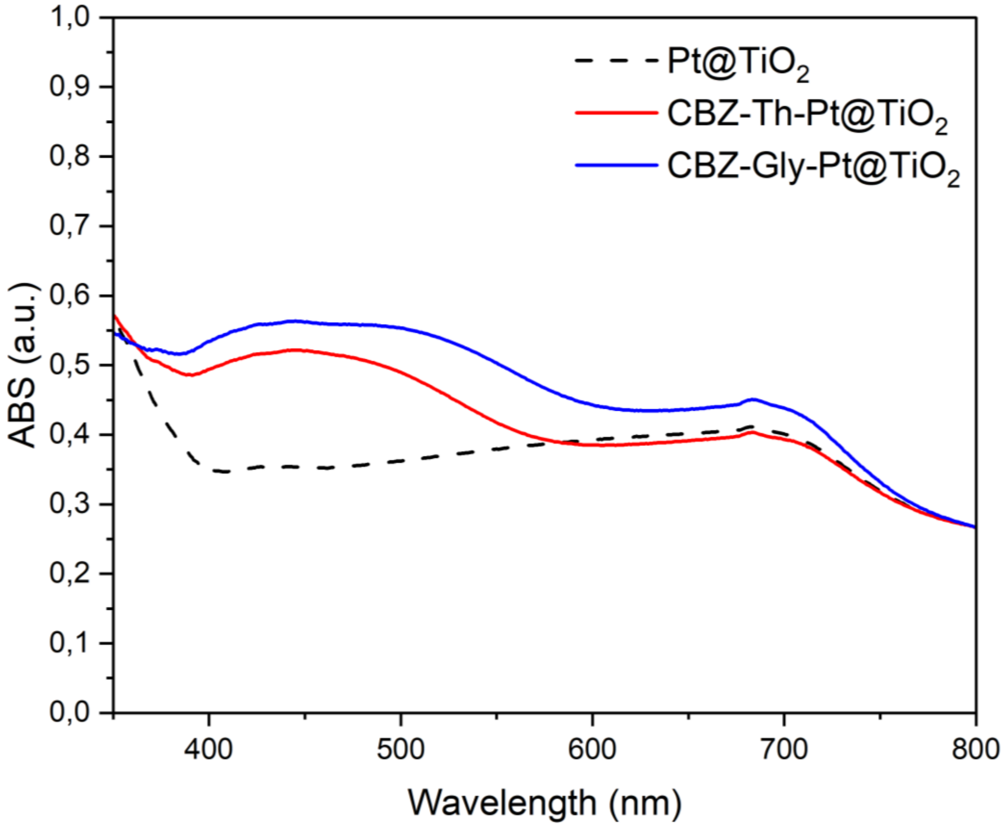

Figure S1. UV-Vis diffuse reflectance measurements of the dyes adsorbed on Pt@TiO_2.

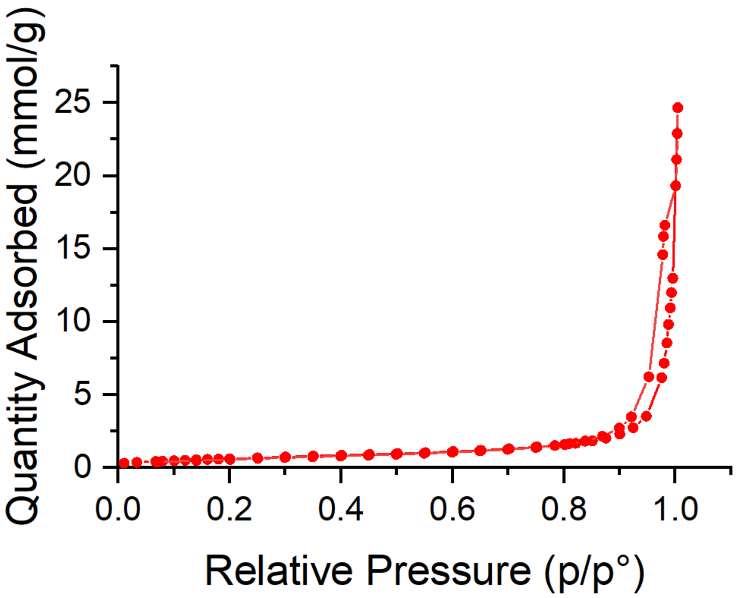

Figure S2. Adsorption–desorption N_2 isotherms measured at 77 K for Pt@TiO_2 sample.
